# *Clostridioides difficile* infections in the intensive care unit: a monocentric cohort study

**DOI:** 10.1007/s15010-020-01413-8

**Published:** 2020-03-24

**Authors:** Rebeca Cruz Aguilar, Jon Salmanton-García, Jonathan Carney, Boris Böll, Matthias Kochanek, Nathalie Jazmati, Oliver A. Cornely, Maria J. G. T. Vehreschild

**Affiliations:** 1 Department I of Internal Medicine, Center for Integrated Oncology Aachen Bonn Cologne Duesseldorf, University of Cologne, Cologne, Germany; 2 Department of Internal Medicine, Zentrum für Innere Medizin, Infectious Diseases, University Hospital Frankfurt, Goethe University Frankfurt, Frankfurt, Germany; 3 German Centre for Infection Research (DZIF), Partner Site Bonn-Cologne, Cologne, Germany; 4 Cologne Excellence Cluster on Cellular Stress Responses in Aging-Associated Diseases (CECAD), University of Cologne, Cologne, Germany; 5 Center for Integrated Oncology CIO Köln/Bonn, Medical Faculty, University of Cologne, Cologne, Germany; 6 Institute for Medical Microbiology, Immunology and Hygiene, University of Cologne, Cologne, Germany; 7 Labor Dr. Wisplinghoff, Cologne, Germany; 8 Clinical Trials Centre Cologne (ZKS Köln), University of Cologne, Cologne, Germany; 9 Center for Molecular Medicine Cologne (CMMC), University of Cologne, Cologne, Germany

**Keywords:** *Clostridioides difficile*, Intensive care, Diarrhea, Morbidity, Mortality

## Abstract

**Introduction:**

Patient-level data from *Clostridioides difficile* infections (CDI) treated in an intensive care setting is limited, despite the growing medical and financial burden of CDI.

**Methods:**

We retrospectively analyzed data from 100 medical intensive care unit patients at the University Hospital Cologne with respect to demography, diagnostics, severity scores, treatment, and outcome. To analyze factors influencing response to treatment and death, a backward-stepwise multiple logistic regression model was applied.

**Results:**

Patients had significant comorbidities including 26% being immunocompromised. The mean Charlson Comorbidity Index was 6.3 (10-year survival rate of 2.25%). At the time of diagnosis, the APACHE II was 17.4±6.3 (predicted mortality rate of 25%), and the ATLAS score was 5.2±1.9 (predicted cure rate of 75%). Overall, 47% of CDI cases were severe, 35% were complicated, and 23% were both. At least one concomitant antibiotic was given to 74% of patients. The cure rate after 10 and 90 days was 56% and 51%, respectively. Each unit increment in APACHE II score was associated with poorer treatment response (OR 0.931; 95% CI 0.872–0.995; *p* = 0.034). Age above 65 years was associated with death (OR 2.533; 95% CI 1.031–6.221; *p* = 0.043), and overall mortality at 90 days was 56%.

**Conclusions:**

CDI affects a high-risk population, in whom predictive scoring tools are not accurate, and outcomes are poor despite intensive treatment. Further research in this field is warranted to improve prediction scoring and patient outcomes.

**Electronic supplementary material:**

The online version of this article (10.1007/s15010-020-01413-8) contains supplementary material, which is available to authorized users.

## Background

*Clostridioides difficile,* a ubiquitous Gram-positive, spore-forming anaerobic bacillus, remains the leading cause of health-care-associated infectious diarrhea in hospitalized patients [1–7], primarily affecting elderly patients with significant comorbidities and previous antibiotic exposure [8–12]. This represents a significant clinical and economic burden and is associated with high rates of morbidity and mortality, although the exact attributable effect of CDI on mortality, particularly in ICU patients, is not yet clear [13–19]. In addition, recurrence rates are as high as 20–30% after standard treatment with metronidazole or vancomycin [20].

Patients hospitalized in intensive care units (ICU) are at a higher risk of infection with CDI compared to patients in standard care wards [21]. Due to the frequent use of broad-spectrum antibiotics, the lack of evidence concerning treatment options for intubated patients, and the severe underlying comorbidities of ICU patients, their treatment is particularly challenging and often associated with a prolonged length of hospital stay (LoS), as well as an increased mortality rate. In a meta-analysis performed by Karanika et al., excess LoS was 18 days in patients with CDI, and the mortality rate was 32% compared to 24% among non-CDI patients [21, 22]. Despite the growing clinical and economic burden, epidemiological assessment of this population remains incomplete and mostly limited to the assessment of incidence of CDI within the ICU [21, 23]. In Europe, studies have been published focusing on incidence, particular risk factors, guideline adherence and management, but none have reported epidemiological data or factors associated with outcomes [24–26]. These data are, however, not fully suitable for a complete clinical understanding of CDI in the ICU. Such data are urgently needed to prepare clinical trials for alternative treatment options for patients unable to swallow oral medication.

The purpose of this analysis was therefore to perform a comprehensive assessment of patient characteristics and clinical outcomes of CDI among ICU patients, as well as potential predictors of response to treatment and death.

## Methods

At the University Hospital Cologne, data from 100 consecutive adult medical ICU patients (≥ 18 years), who were diagnosed with CDI between 01/2013 and 12/2017, were collected and analyzed retrospectively. Inclusion criteria were diagnosis of CDI during ICU stay or within 72 h prior to ICU admission. The diagnosis was based on the diagnostic guidelines of the European Society of Clinical Microbiology and Infectious Diseases (ESCMID), requiring the presence of diarrhea (defined as ≥ 3 unformed bowel movements (UBM)/24 h) plus an enzyme immunoassay (EIA) detecting glutamate dehydrogenase (GDH) and a positive EIA for toxin A or B [27].

CDI was defined as severe if patients had a fever of > 38.5° C, a white blood cell count of ≥ 15 × 10^3^/µl, or a creatinine of ≥ 1.5 times the baseline level. CDI was recorded as complicated if at least one of the following occurred: hypotension requiring vasopressors, ICU admission for a complication of CDI, ileus leading to placement of a nasogastric tube, toxic megacolon, colonic perforation, or colectomy.

Patients were classified as immunocompromised if one of the following features were present: neutropenia, (defined as < 500 neutrophils/µl), previous allogeneic stem cell transplant, inherited severe immunodeficiency (such as chronic granulomatous disease or severe combined immunodeficiency), prolonged use of corticosteroids at a mean minimum dose of 0.3 mg/kg/day of prednisone equivalent for at least 3 weeks within the last 3 months, or treatment during the past 90 days with other recognized T-cell immunosuppressants, such as cyclosporine, TNF-alpha blockers, nucleoside analogs, or specific monoclonal antibodies like alemtuzumab.

The following patient characteristics at diagnosis were registered: age, comorbidities, antibiotic use (including for treatment of diseases other than CDI), kidney and liver function, impairment of the immune system, as well as severity of disease scores including ATLAS (age, temperature, leukocytes, albumin, systemic antibiotics), Charlson Comorbidity Index, and Acute Physiology And Chronic Health Evaluation II score (APACHE II) [28]. Although the APACHE II score is only validated for use when calculated at admission to ICU, in our study we chose to calculate it at the time of CDI diagnosis to assess its prognostic potential concerning CDI outcomes. The closest value measured (± 3 days) was used for laboratory values missing at baseline.

Response to treatment was defined as having < 3 UBM in 24 h within 10 days after treatment initiation. Otherwise, patients were documented as treatment failures. Recurrence was defined as a new episode of ≥ 3 UBM at any point after response to treatment. The patient was considered to have a sustained clinical cure if there were no episodes of recurrence within 90 days of diagnosis of CDI.

To assess overall and CDI-related mortality, ICU charts were evaluated by a physician.

Statistical analyses were carried out using SPSS, version 25.0, Chicago, IL, USA. Continuous variables were presented as mean ± standard deviation or median and interquartile range, as appropriate, while categorical variables were presented as number and percentages. Student's *t* test was used to compare continuous variables, while a Chi-squared test (*χ*^2^) or Fisher’s exact test was used to analyze the frequency distribution between categorical variables. To analyze factors influencing response to treatment and death, variables with a *p* value below 0.1 in univariate analysis were entered into a backward-stepwise multiple logistic regression model.

For further sensitivity analysis considering factors associated with mortality, we used a multivariate Cox proportional hazards regression model looking at the data at the time of discharge, 30 days after diagnosis of CDI, and 90 days after diagnosis of CDI.

Hazard ratios (HRs) and 95% CIs were calculated based on the respective coefficients for these predictors. The assumption of proportional hazards was tested based on Schoenfeld residuals. All statistical tests were two-tailed, and a *p* value of < 0.05 was considered to be statistically significant.

### Ethical considerations

Ethical approval was obtained from the local ethics committee (Refs. 17–113). Informed consent was waived due to the retrospective setting.

## Results

### Patient characteristics

Data on baseline patient characteristics are detailed in Table 1. To avoid undue repetition, percentages have not been shown for numbers involving the 100 patient cohort. Median age was 71 years (IQR 63–77). A total of 26 patients were considered immunocompromised. The median number of hospitalizations in the 6 months prior to CDI diagnosis was 2 (IQR 1.0–2.3) and the median duration of hospitalization was 36 days (IQR 20–61).

**Table 1:** Patient characteristics

Age (years)—median (IQR)	71.0 (63.0–77.0)
Male—*n* (%)	57 (57.0)
Immunocompromised—*n* (%)	26 (26.0)
Hospitalizations within 3 months prior to diagnosis—median (IQR)	2.0 (1.0–2.3)
Laboratory at diagnosis—mean ± SD	
Creatinine (mg/dl)	1.6 ± 1.3
GFR (ml/min)	61.8 ± 39.8
Bilirubin (mg/dl)	1.1 ± 2.6
AP (U/l)	124.8 ± 127.4
γGT (U/l)	180.9 ± 211.1
ALT (U/l)	36.8 ± 52.4
AST (U/l)	55.8 ± 84.5
Hemodialysis at diagnosis—*n* (%)	15 (15.0)
Mechanical ventilation	65 (65.0)
Duration of hospitalization (days)—median (IQR)	36.0 (20.0–61.0)
General ward	7.0 (1.0–19.0)
Intensive care	18.5 (9.3–36.0)
Comorbidities—*n* (%)	
Cardiovascular diseases	84 (84.0)
Nephrological diseases	53 (53.0)
Pulmonary diseases	48 (48.0)
Gastroenterologic diseases, except from *Clostridium difficile* infection	47 (47.0)
Endocrinologic diseases	42 (42.0)
Hematological/oncological diseases	36 (36.0)
Psychiatric diseases	17 (17.0)
Other	58 (58.0)
Scores—mean ± SD	
Charlson Comorbidity Index	6.3 ± 4.3
APACHE II score	17.4 ± 6.3
ATLAS score	5.2 ± 1.9
Death—*n* (%)	56 (56.0)
Infectious (not CDI)	7 (12.5)
Cardiovascular	4 (7.1)
Malignancy	1 (5.6)
Respiratory	1 (5.6)
Unknown	43 (76.8)

Mean creatinine at diagnosis was 1.6 ± 1.3 mg/dl (upper limit of normal male: 1.1 mg/dl; female: 0.9 mg/dl) and mean glomerular filtration rate (GFR) was 61.8 ± 39.8 ml/min, reflecting decreased kidney function; 15 patients required hemodialysis.

All baseline liver function values were within the normal range except for γGT (180.9 ± 211.1 U/l), which was threefold higher than normal.

Cardiovascular diseases were the most commonly diagnosed comorbidities in this cohort (*n* = 84), followed by kidney diseases (*n* = 53). The mean Charlson Comorbidity Index was 6.3 ± 4.3, reflecting a 10-year overall survival probability of 2.25%. The APACHE II score at the time point of diagnosis was 17.4 ± 6.3, with a predicted mortality rate of 25%; the ATLAS score was 5.2 ± 1.9 with a predicted CDI cure rate of 75%.

### CDI treatment and outcome

Details on CDI severity, treatment, and outcome are shown in Table 2. Overall, 47 cases were classified as severe and 35 as complicated; 23 cases were both severe and complicated. The most common reason for classification as complicated CDI was hypotension requiring vasopressors (*n* = 33, 94.3%); however, in only seven cases (21.2%) was CDI considered the cause of hypotension. A total of four patients were transferred to the ICU with active CDI, all others were first diagnosed with CDI while on the ICU.

**Table 2:** Characteristics of *Clostridium difficile* infections and treatment

Severe CDI—*n* (%)	47 (47.0)
White blood cell count ≥ 15 × 10^3^/μL	31 (66.0)
Creatinine ≥ 1.5 times the baseline level	25 (53.2)
Complicated CDI—*n* (%)	35 (35.0)
Hypotension requiring vasopressors	33 (94.3)
ICU admission for a complication of CDI	6 (18.2)
Ileus leading to placement of a nasogastric tube	0 (0.0)
Toxic megacolon	0 (0.0)
Colonic perforation	0 (0.0)
Colectomy or colostomy	0 (0.0)
First line treatment of CDI—*n* (%)	100 (100.0)
Vancomycin	71 (71.0)
Dosage (mg)	125 (125.0–125.0)
Times/day	4.0 (4.0–4.0)
Days	10.0 (9.0–14.0)
Resolution of diarrhea—*n* (%)	48 (70.8)
Days to resolution of diarrhea	8.0 (5.0–11.0)
Metronidazole p.o	18 (18.0)
Dosage (mg)	400.0 (400.0–500.0)
Times/day	3.0 (2.0–3.0)
Days	10.0 (9.0–11.0)
Resolution of diarrhea—*n* (%)	14 (77.8)
Days to resolution of diarrhea	4.0 (2.0–8.0)
Other*	11 (11.0)
Sequential second-line treatment—*n* (%)	17 (17.0)
Vancomycin–fidaxomicin	5 (50.0)
Vancomycin–metronidazole	3 (30.0)
Metronidazole–vancomycin	2 (20.0)
Vancomycin/metronidazole i.v.–vancomycin	2 (20.0)
Vancomycin/metronidazole i.v.–fidaxomicin	1 (10.0)
Metronidazole p.o.–fidaxomicin	1 (10.0)
Metronidazole p.o.–vancomycin/metronidazole p.o	1 (10.0)
Vancomycin 250 mg–vancomycin 125 mg	1 (10.0)
Vancomycin—vancomycin/metronidazole i.v	1 (10.0)
Sequential third line treatment—*n* (%)	3 (3.0)
Metronidazole p.o.–vancomycin/metronidazole p.o.–vancomycin	1 (33.3)
Vancomycin–fidaxomicin–vancomycin	1 (33.3)
Vancomycin–vancomycin/metronidazole i.v.–vancomycin	1 (33.3)
Concomitant antibiotic treatment not active against *C. difficile*—*n* (%)	74 (74.0)
Piperacillin/tazobactam	40 (40.0)
Meropenem	20 (20.0)
Ciprofloxacin	14 (14.0)
Vancomycin	10 (10.0)
Ampicillin/sulbactam	6 (6.0)
Ceftriaxone	6 (6.0)
Others	29 (29.0)

*Other vancomycin/metronidazole (p.o.) *n* = 1, vancomycin/metronidazole (i.v.) *n* = 6; fidaxomicin *n* = 4

*p.o.* per os, *i.v.* intravenous

At day 10, 51 patients had responded to treatment and 49 had ongoing symptoms. Out of the 49 non-responders, 21 presented a delayed response to treatment (> 10 days), 17 received second-line treatment, 6 were transferred to another hospital during the observational period after day 10 (lost to follow-up) and 5 died during the observational period.

Overall, 74 patients received at least one antibiotic in addition to the antibiotics used to treat CDI. The most frequently used concomitant antibiotic was piperacillin/tazobactam (*n* = 40; 54.1%), followed by meropenem (*n* = 20; 27.0%), ciprofloxacin (*n* = 14; 18.9%) and vancomycin (*n* = 10; 13.5%). The CDI treatment response rate after 10 days was 56.0%. Taking recurrences (5.0%) into account, a sustained cure rate of 51.0% at day 90 was determined. Median time to resolution of diarrhea was 7 days (IQR 0–16).

Oral vancomycin alone was the regime used most frequently as first-line treatment (*n* = 71). In these cases, 48 reported a response to treatment (46.5%) with a median duration of treatment of 10 days (IQR 9.0–14.0). Median time to resolution of diarrhea was 8.0 days (IQR 5.0–11.0). A second treatment was documented in 17 patients. In this group, fidaxomicin was the most frequently prescribed substance (*n* = 7; 41.2%), followed by vancomycin alone (*n* = 5; 29.4%) and metronidazole alone (*n* = 3; 17.6%).

We identified a total of 20 patients who were unable to swallow and therefore required metronidazole i.v. treatment or treatment through an enteral feeding tube. Of the 18 patients receiving initial monotherapy with metronidazole, 11 presented with severe CDI.

Mortality occurring within 90 days of diagnosis was 56%. Only one death was classified as CDI related. Median time to death was 81 days (IQR 29.5–265.8). Overall, 16 patients (28.6%) died before and 40 (71.4%) after discharge.

Multivariate analysis was used to assess risk factors for treatment failure (Supplementary Table 1). The only statistically significant factor discovered was that each unit increment in APACHE II score was associated with a poorer response to treatment (OR 0.931; 95% CI 0.872–0.995; *p* = 0.034).

Supplementary Table 2 contains data from the logistic regression assessing factors associated with mortality. Age > 65 years had a statistically significant association with risk of death (OR 2.787; 95% CI 1.163–6.676; *p* = 0.021). An association with death was also seen on univariate analysis with each unit increment of the Charlson Comorbidity Index, but after multivariate analysis this was not statistically significant.

Cox logistic regressions with death as the dependent variable were performed looking at day of discharge, 30 days after diagnosis of CDI, and 90 days after diagnosis of CDI, but no statistically significant associations were found.

## Discussion

Our study confirms that treatment of CDI in the critically ill patient remains a challenge at multiple levels. In our population, response to treatment after a 10-day-antibiotic course was 56%, which is significantly lower than the 62–75% response rate reported in other ICU cohorts. [18, 24, 29]. The median time to resolution of diarrhea was 7 days (IQR 0–16). This is substantially longer than the 3 days reported for non-ICU patients [30, 31]. This low response rate and prolonged time to response may be due to the high rate of concomitant antibiotics and other treatments frequently used in the ICU that can cause diarrhea as a side effect, such as enteral nutrition. In 74% of cases, at least one concomitant antibiotic was administered. Since discontinuation of concomitant antibiotics is considered a key measure in the treatment of CDI, this ongoing antibiotic exposure during CDI treatment represents a major challenge with respect to sustained cure. It is interesting, however, that in our study concomitant antibiotics were not associated with poorer response to treatment, which may indicate that our study was underpowered concerning this association.

The higher proportion of immunocompromised patients (26%) in this analysis than in other cohorts may also have contributed to the difficulties in achieving an adequate treatment response. This likely reflects the high rate of cancer patients treated at the University Hospital Cologne [12, 14, 23].

It seems contradictory that metronidazole was associated with the shortest time to response, as its inferiority to vancomycin has been previously demonstrated [32]. However, it is likely that those who received metronidazole had less severe disease and therefore would naturally have shorter response times than severely ill patients. Initially, it may be surprising to see that patients receiving metronidazole alone received oral treatment instead of intravenous treatment. Again, it is likely that those prescribed metronidazole had less severe infections than those on other treatments, and therefore would be more appropriate to be given oral metronidazole.

Overall mortality in this analysis was 56%, which appears high. Since it is difficult to assess the specific impact of CDI on the death of patients with complex underlying diseases, the attributable impact of CDI on this result remains unclear. In other cohorts, crude mortality rates ranged from 13 to 37% for non-ICU patients, [3, 33] and were 28–40% in the ICU [3, 18, 19, 21]. As only one death (1/56, 1.79%) was attributed to CDI, it is unlikely that the effects of the CDI treatments would have affected the crude mortality rate in our study. The high proportion of immunocompromised patients in our cohort may be a contributing factor to this discrepancy.

We tested a number of scoring systems in the hope of finding one which would be helpful for predicting outcomes for future treatments. It is interesting to see that each unit increment of APACHE II predicted failure of first-line treatment, but did not predict increased mortality. This may be due to documenting APACHE II at diagnosis of CDI instead of ICU admission. It is likely that by then, many of the values integrated into the APACHE II score would have already been corrected by intensive care treatment, resulting in minimized inter-patient differences in the score. The Charlson Comorbidity Index was not successful in predicting survival, likely as it focuses on chronic underlying diseases and not on the acute setting. The ATLAS score was not accurate in predicting cure rate, which could be due to the fact that this score was validated in a less ill, more stable population, and was not specifically designed for ICU patients [28].

For future studies, it may be of use to additionally document the APACHE score at transfer to the ICU and to add documentation of the Simplified Acute Physiology Score (SAPS) II and Sequential Organ Failure Assessment (SOFA) score, which may succeed in differentiating the acute clinical status of a patient in intensive care more effectively.

Our analysis is limited by its monocentric retrospective documentation and the low sample size. Data collection was conducted in an academic hospital with a high percentage of hematology, oncology, and otherwise immunocompromised patients. Depending on the thematic focus of other sites, underlying diseases may vary substantially. The decision to consider response to treatment as having less than 3 UBM in a 24 h period within the first 10 days after treatment initiation is likely to have artificially lowered the response rate, as mentioned above. This is due to the fact that patients in ICUs can have diarrhea for reasons other than CDI, which is neither well captured nor accounted for in our study. Treating a significant reduction in UBM as an alternative criteria for response to treatment may be an alternative approach to this issue. Furthermore, retrospective documentation limits follow-up possibilities. Future conduct of a prospective observational multicenter study may help to reduce these biases.

## Electronic supplementary material

Below is the link to the electronic supplementary material.

Supplementary file1 (DOCX 19 kb)
