# Supplementary material for: Clostridioides difficile infections in the intensive care unit: a monocentric cohort study
Source: Infection. 2020 Mar 24;48(3):421–7. doi: 10.1007/s15010-020-01413-8 (PMC7256083; doi:10.1007/s15010-020-01413-8)
Supplement: Supplementary file 1 — Supplementary file1 (DOCX 19 kb) [file 15010_2020_1413_MOESM1_ESM.docx]

**Supplement**

Supplementary Table 1: Logistic regression assessing factors influencing response to first treatment of CDI

| Dependant variable:  Response to first line treatment | Univariate | | | | Multivariate | | | |
| --- | --- | --- | --- | --- | --- | --- | --- | --- |
|  | p-value | OR | 95 % CI | | p-value | OR | 95 % CI | |
| Age |  |  |  |  |  |  |  |  |
| ≤65 | - |  |  |  |  |  |  |  |
| >65 | 0.476 | 0.730 | 0.308 | 1.732 |  |  |  |  |
| Concomitant antibiotic treatment | 0.509 | 0.735 | 0.295 | 1.832 |  |  |  |  |
| First line treatment of CDI |  |  |  |  |  |  |  |  |
| Fidamoxicin | - |  |  |  |  |  |  |  |
| Metronidazol p.o. | 0.905 | 1.167 | 0.094 | 14.518 |  |  |  |  |
| Vancomycin p.o. | 0.293 | 0.289 | 0.029 | 2.918 |  |  |  |  |
| Vancomycin/Metronidazol p.o. | 1.000 | - | - | - |  |  |  |  |
| Vancomycin/Metronidazol i.v. | 0.748 | 1.667 | 0.074 | 37.728 |  |  |  |  |
| Creatinine |  |  |  |  |  |  |  |  |
| ≤1.5 mg/dl | - |  |  |  |  |  |  |  |
| >1.5 mg/dl | 0.683 | 1.190 | 0.515 | 2.750 |  |  |  |  |
| Haemodialysis | 0.822 | 0.881 | 0.293 | 2.650 |  |  |  |  |
| Mechanical ventilation | 0.153 | 0.538 | 0.230 | 1.260 |  |  |  |  |
| Immunosuppression | 0.840 | 1.098 | 0.445 | 2.708 |  |  |  |  |
| Severe CDI | 0.498 | 1.316 | 0.595 | 2.910 |  |  |  |  |
| Complicated CDI | 0.500 | 0.752 | 0.329 | 1.719 |  |  |  |  |
| Severe and Complicated CDI | 0.674 | 0.818 | 0.321 | 2.083 |  |  |  |  |
| Severe or Complicated CDI | 0.694 | 1.175 | 0.527 | 2.620 |  |  |  |  |
| APACHE II score | 0.034* ᵃ | 0.931 | 0.872 | 0.995 | 0.017* | 0.916 | 0.852 | 0.984 |
| 0-15 | - |  |  |  |  |  |  |  |
| 16-25 | 0.073° | 0.457 | 0.194 | 1.075 | 0.055 | 0.387 | 0.147 | 1.021 |
| >25 | 0.200 | 0.417 | 0.109 | 1.590 | 0.086 | 0.286 | 0.068 | 1.195 |
| ATLAS score | 0.250ᵇ | 0.882 | 0.713 | 1.092 |  |  |  |  |
| 0-5 | - |  |  |  |  |  |  |  |
| 6-10 | 0.067° | 0.469 | 0.209 | 1.054 | 0.198^ | 0.542^ | 0.213^ | 1.378^ |
| Charlson Comorbidity Index | 0.982ᶜ | 0.999 | 0.911 | 1.095 |  |  |  |  |
| 0-6 | - | - | - | - |  |  |  |  |
| >6 | 0.325 | 0.667 | 0.298 | 1.494 |  |  |  |  |

CDI=*Clostridioides difficile* infection, ATLAS= Age, Temperature, Leukocytosis, Albumin, and Systemic concomitant antibiotic use, APACHE= Acute Physiology And Chronic Health Evaluation p.o.: per os; i.v.: intravenous

* p-value ≤0.05

° p-value ≤0.10

^ Out of the model

ᵃ APACHE Score used as continuous variable

ᵇ ATLAS Score used as continuous variable

ᶜ Charlson Comorbidity Index used as continuous variable

Supplementary Table 2: Logistic regression assessing factors influencing death

| Dependant variable: Death | Univariate | | | | Multivariate | | | |
| --- | --- | --- | --- | --- | --- | --- | --- | --- |
|  | p-value |  |  |  | p-value | OR | 95 % CI | |
| Age |  |  |  |  |  |  |  |  |
| ≤ 65 | - |  |  |  |  |  |  |  |
| > 65 | 0.021* | 2.787 | 1.163 | 6.676 | 0.043 * | 2.533 | 1.031 | 6.221 |
| Concomitant antibiotic treatment | 0.509 | 0.735 | 0.295 | 1.832 |  |  |  |  |
| Treatment of CDI #1 |  |  |  |  |  |  |  |  |
| Fidamoxicin | - |  |  |  |  |  |  |  |
| Metronidazol p.o. | 0.483 | 0.417 | 0.036 | 4.813 |  |  |  |  |
| Vancomycin | 0.445 | 0.406 | 0.040 | 4.097 |  |  |  |  |
| Vancomycin/Metronidazol p.o. | 1.000 | - | - | - |  |  |  |  |
| Vancomycin/Metronidazol i.v. | 0.437 | 0.333 | 0.021 | 5.329 |  |  |  |  |
| Creatinine |  |  |  |  |  |  |  |  |
| ≤ 1.5 mg/dl | - |  |  |  |  |  |  |  |
| > 1.5 mg/dl | 0.095° | 2.091 | 0.880 | 4.968 | 0.192^ | 1.826^ | 0.739^ | 4.513^ |
| Haemodialysis | 0.735 | 1.213 | 0.396 | 3.709 |  |  |  |  |
| Mechanical ventilation | 0.273 | 1.588 | 0.694 | 3.635 |  |  |  |  |
| Immunosuppression | 0.840 | 1.098 | 0.445 | 2.708 |  |  |  |  |
| Severe CDI | 0.784 | 1.117 | 0.506 | 2.466 |  |  |  |  |
| Complicated CDI | 0.866 | 1.074 | 0.469 | 2.461 |  |  |  |  |
| Severe and Complicated CDI | 0.954 | 1.028 | 0.402 | 2.629 |  |  |  |  |
| Severe or Complicated CDI | 0.694 | 1.175 | 0.527 | 2.620 |  |  |  |  |
| APACHE II score | 0.322 ᵃ | 1.033 | 0.969 | 1.101 |  |  |  |  |
| 0-15 | - |  |  |  |  |  |  |  |
| 16-25 | 0.335 | 1.510 | 0.653 | 3.492 |  |  |  |  |
| >25 | 0.166 | 2.788 | 0.654 | 11.884 |  |  |  |  |
| ATLAS score | 0.158 ᵇ | 1.170 | 0.941 | 1.454 |  |  |  |  |
| 0-5 | - |  |  |  |  |  |  |  |
| 6-10 | 0.312 | 1.517 | 0.676 | 3.403 |  |  |  |  |
| Charlson Comorbidity Index (CCI) | 0.035* ᶜ | 1.118 | 1.008 | 1.240 | 0.063 | 1.101 | 0.995 | 1.219 |
| 0-6 | - |  |  |  |  |  |  |  |
| >6 | 0.286 | 1.559 | 0.689 | 3.527 |  |  |  |  |

CDI=*Clostridioides difficile* infection, ATLAS= Age, Temperature, Leukocytosis, Albumin, and Systemic concomitant antibiotic use, APACHE=Acute Physiology and Chronic Health Evaluation

* p-value ≤0.05

° p-value ≤0.10

^ Out of the model

ᵃ APACHE Score used as continuous variable

ᵇ ATLAS Score used as continuous variable

ᶜ Charlson Comorbidity Index used as continuous variable
